# Recent Trends in the Pharmacological Treatment of Chronic Migraine: A Systematic Review of Randomized Controlled Trials

**DOI:** 10.7759/cureus.95602

**Published:** 2025-10-28

**Authors:** Jitender Sharma, Rahul Soni, Santosh Singh

**Affiliations:** 1 Neurology, Base Hospital Delhi Cantt, New Delhi, IND; 2 Internal Medicine, Armed Forces Medical College, Pune, IND

**Keywords:** chronic migraine, headache, migraine, migraine disorders, pharmacological treatment

## Abstract

Chronic migraine is one of the debilitating neurological conditions affecting the lives of several patients. This systematic review aimed to explore the recent trends in the pharmacological treatment of chronic migraine. We conducted this systematic review following the Preferred Reporting Items for Systematic Reviews and Meta-Analysis guidelines. The literature search encompassed extensive databases such as PubMed, ScienceDirect, and Cochrane Central Register of Controlled Trials. The analysis included studies published from 2014 to 2024, and the quality of the included studies was evaluated using the appropriate tools according to the study design. The synthesis and data analysis included a summary of study characteristics, interventions, outcomes measured, and main study results/conclusions. Sample sizes in the included studies ranged from 1,072 to 2,436 participants. The most common pharmacological treatments used for chronic migraine were eptinezumab, onabotulinumtoxinA, and galcanezumab, which emerged from 14 quality randomized controlled trials. Studies observed significant improvements across various metrics, including headache frequency, severity, quality of life measures, and patient-reported outcomes. The Headache Impact Test-6 score, monthly migraine headache days, Migraine-Specific Quality-of-Life score, and Migraine Disability Assessment score were particularly useful in quantifying the treatment’s effectiveness. This systematic review concludes the effectiveness and safety of newer treatments such as eptinezumab, galcanezumab, and onabotulinumtoxinA in managing chronic migraine, illustrating notable improvements in both clinical outcomes and quality of life for patients.

## Introduction and background

Chronic migraine is characterized by headaches that occur for 15 or more days each month for a minimum duration of three months, which significantly impacts the quality of life of patients and places a considerable strain on healthcare systems. Recent advancements in pharmacological treatments have shifted the paradigm of chronic migraine management, focusing on targeted therapies with improved efficacy and tolerability. Traditional preventive treatments, such as topiramate and onabotulinumtoxinA, have long been utilized, with substantial evidence supporting their effectiveness in reducing monthly migraine days (MMDs) and improving patient-reported outcomes. OnabotulinumtoxinA, in particular, has demonstrated long-term safety and efficacy in real-world settings, making it a cornerstone therapy for chronic migraine prevention [1,2].

In recent years, calcitonin gene-related peptide (CGRP) monoclonal antibodies (mAbs) have revolutionized chronic migraine treatment by offering a migraine-specific mechanism of action. These agents, including erenumab, galcanezumab, fremanezumab, and eptinezumab, have shown significant reductions in MMDs and medication overuse compared to placebo in randomized controlled trials (RCTs). Their favorable safety profiles and convenient dosing schedules have further enhanced their appeal as preventive options [3,4]. Additionally, small-molecule CGRP receptor antagonists (gepants) such as rimegepant and atogepant provide oral alternatives for both acute and preventive treatment of migraine [5]. Comparative studies suggest that newer therapies such as CGRP mAbs may offer superior tolerability and efficacy compared to traditional options such as topiramate [3,4].

Despite these advancements, challenges remain in optimizing treatment strategies for patients with refractory chronic migraine or comorbid conditions. Ongoing research continues to explore head-to-head comparisons of these therapies and their long-term effects on patient outcomes. The evolving landscape of chronic migraine pharmacological treatment underscores the importance of individualized approaches to maximize therapeutic benefits while minimizing adverse effects.

Recent advancements in the pharmacological treatment of chronic migraine have significantly transformed management strategies, primarily through the introduction of CGRP pathway interventions. mAbs targeting CGRP, such as erenumab, galcanezumab, fremanezumab, and eptinezumab, have emerged as groundbreaking options for chronic migraine prevention. These therapies have demonstrated substantial efficacy in reducing MMDs and improving overall quality of life for patients. In clinical trials, CGRP mAbs have shown reductions in MMDs that are at least comparable, if not superior, to traditional preventive treatments such as topiramate and onabotulinumtoxinA, with a favorable safety profile characterized by minimal adverse effects and high tolerability [6,7].

The mechanism of action for these mAbs involves blocking the CGRP receptor or inhibiting CGRP itself, which plays a crucial role in migraine pathophysiology by promoting neurogenic inflammation and pain signalling [8,9]. Studies have indicated that these treatments not only reduce headache frequency but also significantly decrease associated symptoms such as photophobia and phonophobia, providing a more comprehensive approach to migraine management [10]. Furthermore, small-molecule CGRP receptor antagonists (gepants), including ubrogepant and rimegepant, offer additional options for both acute and preventive treatment of migraines, expanding the therapeutic arsenal available to clinicians [11].

Clinical data from large-scale RCTs have reinforced the efficacy of these new agents. For example, patients who received galcanezumab experienced an average decrease of 4.8 MMDs in comparison to the placebo [12]. Moreover, real-world studies support these findings, indicating that CGRP mAbs effectively address treatment-resistant chronic migraines and improve patient adherence due to their favorable side effect profiles [6]. As research continues to evolve, the integration of these novel therapies into clinical practice underscores the importance of personalized treatment strategies aimed at optimizing outcomes for individuals suffering from chronic migraine. The present systematic review aimed to explore the recent trends in the pharmacological treatment of chronic migraine.

## Review

Methodology

Preferred Reporting Guidelines

This systematic review followed the 2020 Preferred Reporting Items for Systematic Reviews and Meta-Analyses (PRISMA) guidelines for transparent and comprehensive reporting [13].

Search Sources and Strategy

We searched databases such as PubMed, ScienceDirect, and Cochrane Central Register of Controlled Trials using a combination of Medical Subject Headings and free-text keywords. The primary search terms were “chronic migraine,” “headache,” “migraine disorders,” and “pharmacological treatment.” These keywords were connected using Boolean operators “AND” and “OR,” and were further refined through the use of synonyms and related terms. Some of the search strings used were “Chronic Migraine” OR “Migraine Disorders” OR “Migraine” OR “Headache Disorders” OR “Headache” OR “Recurrent Headache” AND “Drug Therapy” OR “Pharmacological Treatment” OR “Pharmacotherapy” OR “Medication” OR “Pharmacologic Therapy” OR “Pharmacological Intervention” OR “Drug Treatment” OR “Drug Therapy.”

Screening and Study Selection

Studies published from 2014 to 2024 were included in this systematic review. A thorough pre-screening was performed to ensure the validity and dependability of the literature selection procedure. Two separate researchers conducted this pre-screening, and a third reviewer resolved any disagreements. The abstract and title of every study were carefully reviewed to determine their applicability to the goals of the investigation. The relevant outcome estimates presented in each study were extracted by carefully examining the complete text of the identified studies. By using this exacting approach, we hoped to uphold a high degree of methodological accuracy and integrity throughout the data gathering process, providing a strong basis for the analysis and synthesis of findings that followed.

Inclusion criteria included only RCTs having full text published in the English language that investigated pharmacological treatment for chronic migraine in adult patients (≥18 years) with a sample size of >1,000 patients.

We excluded pediatric patients, studies conducted on episodic migraine, studies published in languages other than English, observational studies, retrospective studies, case studies, commentaries, editorials and short communications, abstracts, literature reviews, systematic reviews and meta-analyses, and studies conducted on animals.

Data Extraction and Synthesis

Data extraction and synthesis were performed after appropriate screening of the studies based on the inclusion and exclusion criteria. The extracted data included publication year, country, study design, sample size, treatment interventions, outcomes, and main findings of the studies. The collected data were presented as findings of this systematic review after analysis.

Quality Assessment of the Studies

The selected studies were assessed for quality assessment using the Cochrane Risk-of-Bias tool for randomized trials (RoB 2) [14]. Two authors independently assessed the quality of the studies, and any disagreements were resolved by consensus discussion. Only studies that met the quality appraisal criteria were finalized for inclusion in this systematic review.

Results

**Figure 1:**
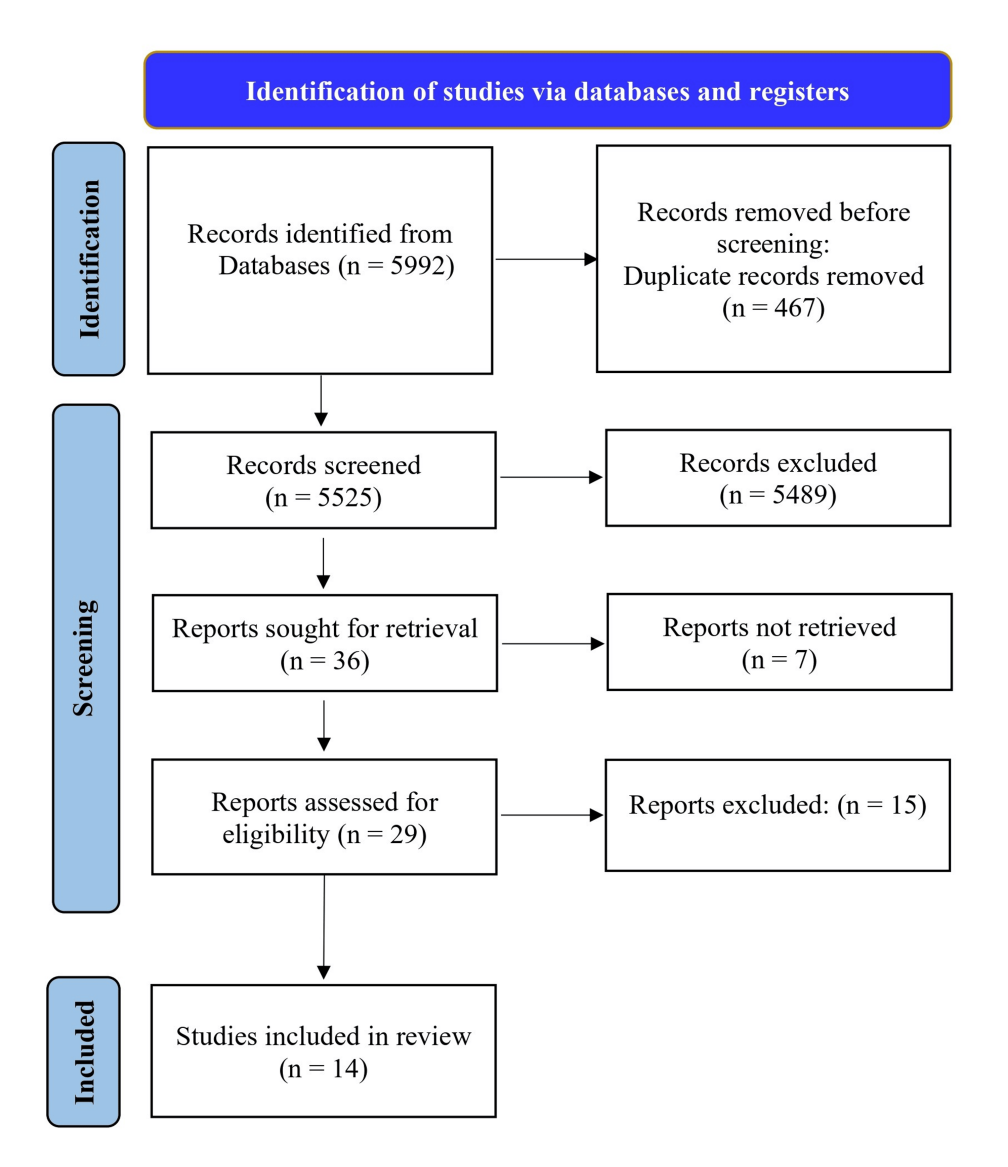
Preferred Reporting Items for Systematic Reviews and Meta-Analyses flowchart.

Our initial search identified 5,992 studies from different databases. In total, 5525 records were screened after initial exclusion of the studies. Following an assessment of the titles and abstracts, 36 articles were selected for further consideration. Following that, seven studies were eliminated based on the inclusion criteria. We evaluated 29 studies according to the established inclusion and exclusion criteria. Finally, we selected 14 studies because of the non-availability of some data in other studies. The procedure for selecting the studies is illustrated in the PRISMA study selection diagram (Figure 1).

Study Characteristics

Table 1 details a systematic review of recent studies [15-28] focusing on the pharmacological treatment trends for chronic migraine, emphasizing various interventions, study designs, outcomes, and conclusions drawn from the findings. The table includes entries from 14 distinct studies, mostly RCTs conducted primarily in the United States, with some from Germany and the United Kingdom. The sample sizes ranged from 2,436 patients in one study to 1,072 in several others, highlighting a robust aggregation of data.

**Table 1:** Characteristics of the included studies. RCT: randomized controlled trial; CM: chronic migraine; HIT-6: Headache Impact Test-6; MSQ: Migraine-Specific Quality-of-Life Questionnaire; HRQoL: health-related quality of life; MHDs: monthly headache days; MMDs: monthly migraine days; MRRs: migraine responder rates; PGIC: patient global impression of change; TEAEs: treatment-emergent adverse events; MIDAS: migraine disability assessment; AHM: acute headache medication; HCRU: healthcare resource utilization; PI-MBS: patient-identified most bothersome symptom; IV: intravenous

Author (year)	Country	Design	Sample size	Intervention	Outcomes assessed	Summary outcome
Diener et al. (2014) [15]	Germany	RCT	2,436	OnabotulinumtoxinA vs. placebo	Adverse events	Well tolerated up to five treatment cycles (75–260 U every 12 weeks) in chronic migraine
Aurora et al. (2014) [16]	USA	RCT	1,384	OnabotulinumtoxinA vs. placebo	Headache days, migraine days, HIT-6, MSQ	Significant benefits across multiple headache measures; earlier initiation led to better outcomes by week 56
Silberstein et al. (2015) [17]	USA	RCT	1,384	OnabotulinumtoxinA vs. placebo	Headache days, HIT-6	Reduced headache-day frequency and improved HIT-6 scores in chronic migraine patients
Lipton et al. (2016) [18]	USA	RCT	1,236	OnabotulinumtoxinA vs. placebo	HRQoL (HIT-6, MSQ)	OnabotulinumtoxinA showed superiority in HIT-6 (48 weeks) and MSQ role-restrictive domain (56 weeks)
Matharu et al. (2017) [19]	UK	RCT	1,384	OnabotulinumtoxinA vs. placebo	Headache days, severity score, HIT-6	Lower severe headache days, reduced severity, and improved HIT-6 at week 24
Detke et al. (2018) [20]	UK	RCT	1,117	Galcanezumab vs. placebo	Monthly MHDs	Galcanezumab superior in reducing MHDs; efficacious, safe, and well tolerated
Silberstein et al. (2020) [21]	USA	RCT	1,072	Eptinezumab vs. placebo	Migraine days, MRRs, HIT-6, PGIC	Eptinezumab (100/300 mg IV) showed sustained efficacy and safety over 24 weeks
Lipton et al. (2020) [22]	USA	RCT	1,072	Eptinezumab vs. placebo	MMDs, TEAEs	Reduced MMDs with favorable safety profile; onset of efficacy from day 1
Lipton et al. (2021) [23]	USA	RCT	1,130	Fremanezumab vs. placebo	Headache/migraine days, PGIC, HIT-6, depression	Effective for chronic migraine prevention and reduced headache impact in patients with comorbid depression
Ford et al. (2021) [24]	USA	RCT	1,113	Galcanezumab vs. placebo	MSQ, MIDAS	Improved functioning and disability with clinically significant change
Cowan et al. (2022) [25]	USA	RCT	1,072	Eptinezumab vs. placebo	AHM use, headache frequency	Reduced headache frequency and AHM use
Tobin et al. (2022) [26]	USA	RCT	1,113	Galcanezumab vs. placebo	MHDs, AHM use, HCRU	Decreased HCRU, AHM misuse, and migraine days requiring acute medication
McAllister et al. (2022) [27]	USA	RCT	1,072	Eptinezumab vs. placebo	Headache days, episodes	Greater reduction in headache days and episodes (100 mg: 11.2%, 300 mg: 12.4%)
Tepper et al. (2024) [28]	USA	RCT	1,072	Eptinezumab vs. placebo	HIT-6, PGIC, PI-MBS	Longer interictal periods, improved HIT-6, PGIC, and symptomology

Each study presents a unique aim and corresponding intervention. For instance, Tepper et al. (2024) [28] aimed to explore the relationship between interictal periods and patient-reported outcomes, finding significant improvements associated with longer interictal durations when treated with eptinezumab. Cowan et al. 2022 [25] conducted a post hoc analysis of PROMISE-2, revealing that eptinezumab correlated with reduced headache frequency and acute headache medication usage, particularly among patients with medication overuse headache (MOH). Tobin et al. (2022) [26] analyzed secondary objectives from the REGAIN study, revealing notable decreases in the overuse of acute headache medications and the utilization of healthcare resources among patients treated with galcanezumab. McAllister et al. (2022) [27] observed significant decreases in headache episode severity and frequency in patients treated with eptinezumab compared to placebo. Key findings from Silberstein et al. (2020) [21] revealed that repeated intravenous eptinezumab doses resulted in sustained reductions in migraine days over 24 weeks, with a favorable safety profile. Diener et al. (2014) [15] assessed the safety and tolerability of onabotulinumtoxinA, reporting that while adverse events were common, they were largely manageable and infrequent. Further studies, such as those by Lipton et al. (2020) and (2021) [22,23], evaluated fremanezumab and the specific effects of eptinezumab, respectively, highlighting significant reductions in headache days and a favorable impact on patient-reported quality of life measures, including Headache Impact Test-6 (HIT-6) and migraine-specific quality of life questionnaires. Other notable findings include significant improvements in functioning and disability associated with galcanezumab (Ford et al., 2021 [24]) and the effectiveness of onabotulinumtoxinA in patients who did not respond to initial treatment cycles (Matharu et al., 2017 [19]).

**Figure 2:**
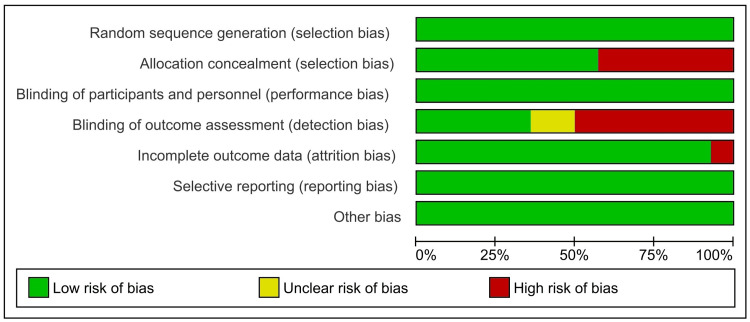
Risk of bias graph of the included studies evaluated using the Risk of Bias 2 tool.

**Figure 3:**
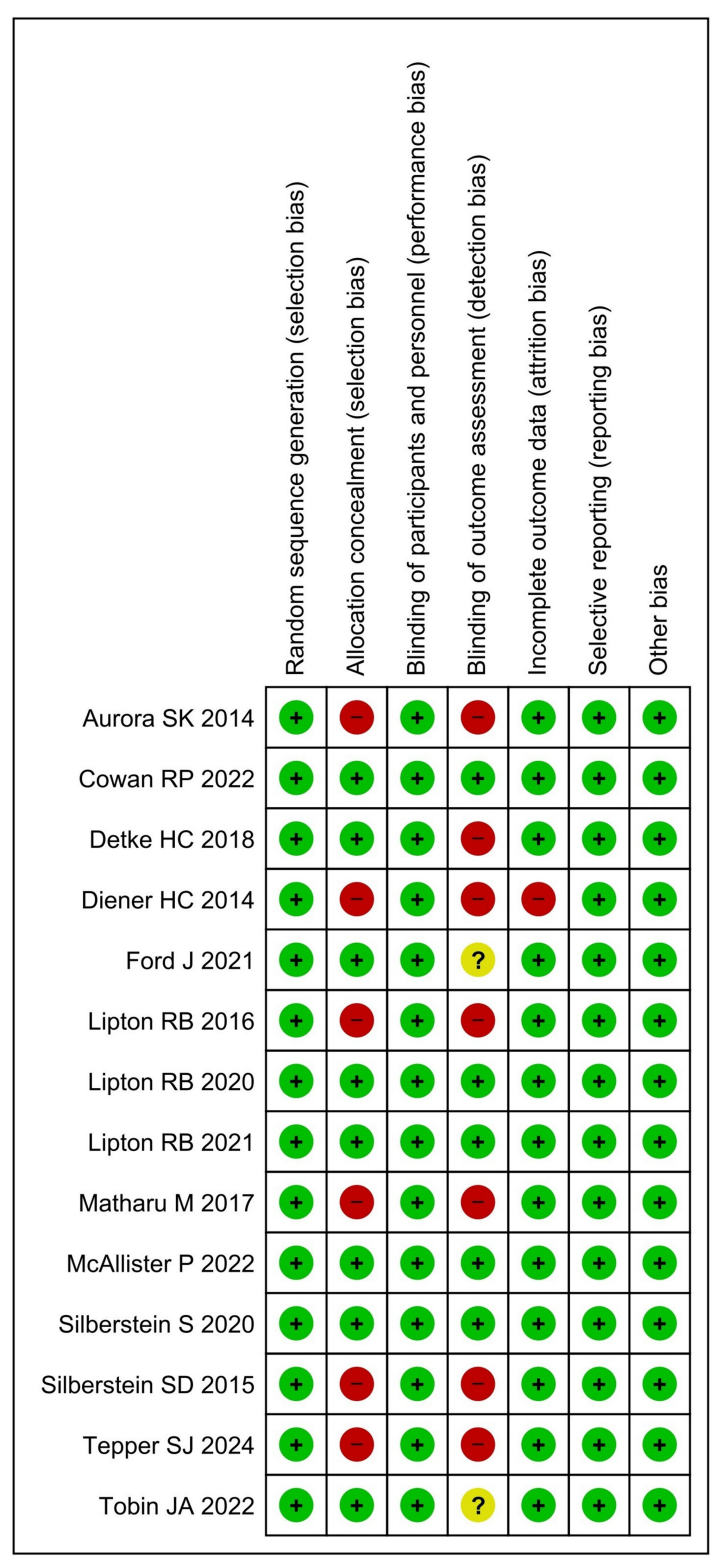
Risk of bias summary of the included studies assessed using the Risk of Bias 2 tool. [15-28].

Figure 2 and Figure 3 present a quality assessment of all included studies using the Cochrane RoB 2, focusing on key indicators of study integrity. Most studies, including those by Diener et al. (2014) [15], Aurora et al. (2014) [16], Silberstein et al. (2015) [17], Lipton et al. (2016, 2020, 2021) [18,22,23], and Matharu et al. (2017) [19], demonstrated a low risk of bias in terms of random sequence generation and blinding of participants and personnel. However, allocation concealment was frequently rated as high risk, particularly in earlier studies (e.g., Diener et al. (2014) [15], Aurora et al. (2014) [16], and Lipton et al. (2016) [18]), whereas later studies, such as those by Silberstein et al. (2020) [21], McAllister et al. (2022) [27], and Cowan et al. (2022) [25], showed improved methodology with a low risk in all or most domains. Blinding of outcome assessment presented variability, with several studies, such as Diener et al. (2014) [15] and Tepper et al. (2024) [28], marked as high risk, while many recent studies achieved low risk or unclear ratings. Incomplete outcome data, reporting bias, and other bias were consistently assessed as low risk across the majority of studies, with the notable exception of Diener et al. (2014) [15], which had high risk in incomplete outcome data. Overall, there appears to be a trend of methodological improvement over time, with more recent trials (2020-2024) generally demonstrating a lower risk of bias across all domains.

Discussion

The present systematic review examining recent trends in the pharmacological treatment of chronic migraine highlights significant findings from multiple RCTs. Notably, eptinezumab was shown to yield superior patient-reported outcomes for individuals with longer interictal periods compared to those with shorter periods, with substantial reductions in the HIT-6 scores and improved overall well-being. Cowan et al. [25] found that eptinezumab also facilitated significant reductions in headache days and acute headache medication use, particularly among patients with MOH.

The findings of our study are consistent with the findings of several previous studies. Eptinezumab has emerged as a promising preventive treatment for chronic migraine, demonstrating significant efficacy and safety across multiple clinical trials. The PROMISE-2 study, a pivotal phase 3 RCT, revealed remarkable outcomes in migraine management [22,29]. Patients receiving eptinezumab experienced substantial reductions in MMDs, with 100 mg and 300 mg doses showing statistically significant decreases of 7.7 and 8.2 days, respectively, compared to 5.6 days in the placebo group [29]. The treatment’s unique advantages include its rapid onset of action, with benefits manifesting as early as the first day after administration. Significantly, the proportion of patients suffering from migraines on any specific day decreased from 58% at baseline to around 50% following eptinezumab treatment [29]. Clinical trials demonstrated impressive migraine responder rates, with 30.9% to 36.9% of patients achieving ≥75% migraine reduction, compared to just 15.6% in the placebo group [29].

Long-term studies have confirmed the sustained effectiveness of eptinezumab, with benefits extending up to 24 weeks and showing particular promise for patients who have failed previous preventive treatments [21,30]. The drug has also demonstrated efficacy across diverse patient populations, including those with episodic and chronic migraine, and patients with MOH [31]. Safety profiles have been consistently favorable, with treatment-emergent adverse events reported in 43.5% to 52% of patients, primarily including upper respiratory tract infections and fatigue [29,32]. The treatment not only reduces migraine frequency but also improves patients’ ability to function, as measured by the HIT-6 [29]. Particularly noteworthy is eptinezumab’s effectiveness for patients with treatment-resistant migraines. Studies have shown significant migraine preventive effects even in adults who have experienced two to four previous preventive treatment failures [30]. This makes eptinezumab a valuable option for patients who have not responded to traditional migraine treatments.

The present findings investigated that onabotulinumtoxinA is effective in reducing headache symptoms compared to placebo for treating chronic migraine. OnabotulinumtoxinA was evaluated across several studies and supported our results, revealing high tolerability and effectiveness in reducing headache frequency and severity. Diener et al. [15] reported that adverse events were infrequent, while Aurora et al. [16] highlighted ongoing improvements in headache-related metrics over multiple treatment cycles, indicating cumulative benefits. Notably, even within non-responder populations, Matharu et al. [19] found that onabotulinumtoxinA could provide significant relief from headache intensity. OnabotulinumtoxinA has emerged as a groundbreaking treatment for chronic migraine, demonstrating significant efficacy and safety across multiple clinical trials. The PREEMPT 2 study revealed remarkable outcomes, showing statistically significant reductions in headache frequency, with patients experiencing a decrease of 9.0 headache days compared to 6.7 days in the placebo group (p < 0.001) [33]. The treatment’s mechanism of action extends beyond traditional muscle relaxation, involving complex neurological interactions. Research indicates that onabotulinumtoxinA inhibits soluble N-ethylmaleimide-sensitive fusion attachment protein receptor (SNARE)-mediated vesicle trafficking, reducing the exocytosis of pro-inflammatory neurotransmitters and neuropeptides in sensory nerves [34]. This innovative approach allows for targeted intervention in chronic migraine pathophysiology.

Long-term studies have consistently supported the treatment’s effectiveness. The PREDICT real-world study demonstrated sustained benefits, with participants experiencing significant improvements in quality of life and headache frequency. Baseline mean headache days decreased from 20.9 to 6.4 days per month, with improvements maintained over two years [35]. Notably, in 2010, it became the first medication specifically approved for chronic migraine prevention [36]. Safety profiles have been remarkably favorable, with few treatment-related adverse events. In clinical trials, only 3.5% of patients discontinued treatment due to side effects [33]. The Canadian PREDICT study further confirmed its safety, with only 2.2% of participants reporting serious treatment-emergent adverse events [35]. The treatment shows particular promise for patients who have failed previous migraine interventions. Multiple studies suggest that onabotulinumtoxinA is especially effective for treatment-resistant chronic migraine patients, offering a valuable option for those with persistent symptoms [37]. Repeated treatments over up to five cycles have demonstrated consistent efficacy and tolerability [38]. Mechanistically, the treatment works by targeting sensory nerve endings in the head and neck, reducing pain signal transmission to the brain. By inhibiting the insertion of pain-sensitive ion channels and decreasing pro-inflammatory neurotransmitter release, onabotulinumtoxinA provides a sophisticated approach to migraine management [34].

OnabotulinumtoxinA demonstrates a sophisticated mechanism of action in treating chronic migraine, extending far beyond traditional muscle relaxation. The treatment operates through complex neurological pathways, primarily by inhibiting SNARE-mediated vesicle trafficking [34]. At the neurochemical level, onabotulinumtoxinA suppresses the exocytosis of pro-inflammatory and excitatory neurotransmitters, including substance P, CGRP, and glutamate from primary afferent fibers [34]. This mechanism significantly reduces nociceptive pain transmission and interrupts both peripheral and central sensitization processes. By decreasing the insertion of pain-sensitive ion channels, such as transient receptor potential cation channel subfamily V member 1, into neuronal membranes, the treatment effectively modulates pain perception [34]. The injection protocol entails focusing on 31-39 locations throughout seven muscles in the head and neck, methodically targeting sensory nerve endings that stem from the trigeminal and cervical ganglia. These nerve endings, typically overactive in migraine patients, are systematically inhibited, consequently reducing pain signals reaching the brain [34,39]. Clinical studies have demonstrated that this approach can significantly decrease headache frequency and severity, with repeated treatments showing sustained efficacy over multiple cycles [38]. Interestingly, the treatment’s effectiveness varies among patients, potentially due to individual differences in underlying pathophysiological mechanisms. Some patients may not respond optimally due to variations in neurotransmitter release mechanisms or the presence of calcium- and SNARE-independent inflammatory mediator secretion pathways [39]. The multifaceted approach of onabotulinumtoxinA includes not only direct analgesic effects but also suppression of myogenic trigger points and interruption of persistent nociceptive barrages that contribute to central sensitization [39]. This comprehensive mechanism positions it as a sophisticated intervention for chronic migraine management, offering hope for patients with treatment-resistant conditions.

The findings of our study demonstrated that treatment with galcanezumab significantly reduced both migraine medication overuse and MMDs compared to placebo in treating patients with chronic migraine. Our results corroborate those of other studies, in which galcanezumab has emerged as a significant pharmacological intervention for chronic migraine, demonstrating substantial efficacy in reducing both MMDs and medication overuse. The REGAIN study, a pivotal phase 3 RCT, found that patients treated with galcanezumab experienced a mean reduction of 4.8 days in monthly migraine headache days (MHDs) for the 120 mg dose and 4.6 days for the 240 mg dose, compared to a reduction of only 2.7 days in the placebo group (p < 0.001) [20]. This finding underscores galcanezumab’s effectiveness in managing chronic migraine symptoms. The mechanism of action for galcanezumab involves its role as a humanized monoclonal antibody targeting CGRP, a key player in migraine pathophysiology. By binding to CGRP, galcanezumab inhibits its interaction with receptors, thereby reducing neurogenic inflammation and pain signalling [40]. This targeted approach has shown promise, particularly in patients with a history of treatment failures, with studies indicating that those who had previously failed one or more preventive treatments experienced significant benefits from galcanezumab [41]. In addition to reducing headache frequency, galcanezumab also significantly decreased medication overuse in chronic migraine patients. A study reported that after 12 months of treatment, patients demonstrated notable improvements not only in MHDs but also in overall quality of life and disability scores, as measured by the HIT-6 and Migraine Disability Assessment [42]. The percentage of patients achieving a ≥50% reduction in MHDs was approximately 57% across treatment groups, highlighting the drug’s potential to address both headache frequency and associated medication overuse effectively. Safety profiles for galcanezumab have been favorable, with most adverse events being mild to moderate and primarily involving injection site reactions [43]. The long-term, open-label extension of the REGAIN study confirmed that galcanezumab remains effective and well-tolerated over extended periods, with high adherence rates among participants [43]. While the findings are promising, some studies suggest variability in response based on baseline characteristics, such as previous treatment failures and severity of migraines at baseline. Patients with higher initial impairment may experience less benefit from treatment compared to those with fewer prior failures [41,42]. Overall, galcanezumab represents a significant advancement in chronic migraine management, offering hope for patients struggling with medication overuse and frequent migraine attacks.

The strength of our systematic review is the inclusion of high-quality RCTs having a large sample size of more than 1,000 patients. The large sample size of the study emphasizes its robustness in terms of its quality and the validity of the results. Limitations of our study include the exclusion of full-text articles that were not retrieved from some databases and the exclusion of articles not in the English language.

## Conclusions

This systematic review is an update of the recent trends in the pharmacological treatment of chronic migraine, highlighting significant findings from several RCTs. Our study found that eptinezumab resulted in a greater reduction in headache days, frequency and severity of headache episodes, and acute medication usage compared to placebo in the treatment of chronic migraine. The present findings investigated that onabotulinumtoxinA is effective in reducing headache symptoms compared to placebo for treating chronic migraine. Fremanezumab is effective for chronic migraine prevention and reduces headache impact in patients with comorbid depression. The findings of our study demonstrated that treatment with galcanezumab significantly reduced both migraine medication overuse and MMDs compared to placebo in treating patients with chronic migraine. Overall, the findings of our systematic review underscore the effectiveness and safety of newer treatments such as eptinezumab, galcanezumab, and onabotulinumtoxinA in managing chronic migraine, illustrating notable improvements in both clinical outcomes and quality of life for patients.
